# Women’s health in the occupied Palestinian territories: Contextual influences on subjective and objective health measures

**DOI:** 10.1371/journal.pone.0186610

**Published:** 2017-10-27

**Authors:** Katie Bates, Tiziana Leone, Rula Ghandour, Suzan Mitwalli, Shiraz Nasr, Ernestina Coast, Rita Giacaman

**Affiliations:** 1 Department of Social Policy, LSE, London, United Kingdom; 2 Department of International Development, LSE, London, United Kingdom; 3 Institute of Community and Public Health, Birzeit University Ramallah, West Bank, Palestine; London School of Hygiene and Tropical Medicine, UNITED KINGDOM

## Abstract

The links between two commonly used measures of health—self-rated health (SRH) and self-reported illness (SRI)–and socio-economic and contextual factors are poorly understood in Low and Middle Income Countries (LMICs) and more specifically among women in conflict areas. This study assesses the socioeconomic determinants of three self-reported measures of health among women in the occupied Palestinian territories; self-reported self-rated health (SRH) and two self-reported illness indicators (acute and chronic diseases). Data were obtained from the 2010 Palestinian Family Health Survey (PFHS), providing a sample of 14,819 women aged 15–54. Data were used to construct three binary dependent variable—SRH (poor or otherwise), and reporting two SRI indicators—general illness and chronic illness (yes or otherwise). Multilevel logistic regression models for each dependent variable were estimated, with individual level socioeconomic and sociodemographic predictors and random intercepts at the governorate and community level included, to explore the determinants of inequalities in health. Consistent socioeconomic inequalities in women’s reports of both SRH and SRI are found. Better educated, wealthier women are significantly less likely to report an SRI and poor SRH. However, intra-oPt regional disparities are not consistent across SRH and SRI. Women from the Gaza Strip are less likely to report poor SRH compared to women from all other regions in the West Bank. Geographic and residential factors, together with socioeconomic status, are key to understanding differences between women’s reports of SRI and SRH in the oPt. More evidence is needed on the health of women in the oPt beyond the ages currently included in surveys. The results for SRH show discrepancies which can often occur in conflict affected settings where a combination of ill-health and poor access to health services impact on women’s health. These results indicate that future policies should be developed in a holistic manner by targeting physical and mental health and well-being in programmes addressing the health needs of women, especially those in conflict affected zones.

## Introduction

Health research and policy efforts focused on women in Low and Middle Income Countries (LMICs) have concentrated on women’s reproductive lives, specifically antenatal care and the spacing and limiting of births. Women’s health beyond reproductive ages in LMICs is generally neglected [1]. We know little about women’s health needs and health service utilization beyond those linked to reproduction [2, 3]. Failure to understand and meet women’s health needs beyond their reproductive years is detrimental to health across the lifecourse, especially given the increasing importance of non-communicable diseases at older ages in LMICs [4]. This neglect is pronounced in areas of protracted conflict such as the occupied Palestinian territory (oPt) where shortages of services and barriers to access make healthcare even more challenging [4].

The oPt is an LMIC with a fragmented, over-burdened and under-resourced health system. Life expectancy at birth is 73 years and there are high levels of poverty and poor nutrition (World Bank 2015). The on-going Israeli military occupation of the oPt has created two administratively separated geographic zones: the Gaza Strip (GS) and the West Bank (WB). The population of the GS bears a heavier burden of structural violence, with restriction of movement, a restricted economy and a resultant lack of access to goods and services alongside exposure to political violence and a fractured health care system [5]. The WB has a more organized health system and is generally less affected by blockades, but receives less aid than the GS. The oPt is a unique comparative conflict-affected context in which to explore the nature of the relationships between SRI and SRH.

Comparative research between the GS and WB has shown that subjective health is heavily influenced by local perceptions of health, both at the neighbourhood level and at the level of wider social networks [6, 7]. Life satisfaction has been found to be higher among Palestinians with strong familial and social networks, despite economic and infrastructural deprivation [8].

Evidence from the oPt shows that women consider the (ill-)health of a family member to take precedence over their own; women’s health is impacted both by their multiple caring responsibilities and a normative understanding that women’s health is less important than the health of others [5, 9]. Inter-related socio-cultural norms of stigma and modesty mean that women’s health-seeking tends to occur only when symptoms of ill-health are present [10, 11]. Low rates of women’s preventative health care (such as cervical cancer screening) have been noted among Palestinian women living in Israel, attributed in part to issues of modesty [12].

Intra-oPt variations in health services and their use are also present with differences in availability, access and utilization of health care between the GS and the WB [13]. Diagnostic testing is frequently limited to specific hospitals or geographic locations and specialized surgery is limited within Palestinian hospitals (such as reconstructive surgery following breast cancer) requiring many women to seek such care in neighboring Israel or Jordan, posing multiple economic and political barriers for Palestinian women’s care-seeking [14].

Self-reported measures are increasingly used to assess health status and needs in LMICs [15, 16]. Self Reported Health (SRH) is usually measured in surveys on a five point scale from very poor to very good. Self-reported illness (SRI) also relies on individual reports, and questions are asked about specific types of disease (chronic or acute) or specific illnesses (such as diabetes and cardiac disease). SRI data collected in surveys is variable and can include data on whether the reported illness was diagnosed by a medical professional, related health-seeking behavior and if medical treatment was or is currently being received. SRI data often include a time dimension which captures, for example, the length of time the illness has been experienced or the time period in which the illness occurred.

Whilst considered a more objective health measure compared to SRH, SRI is based on self- or proxy-reports rather than medical data or diagnosis by a clinical professional.

Both SRH and SRI are independently associated with a wide range of factors (demographic, socioeconomic, education, health behaviours, health knowledge, and context), and the relation between the two indicators provides an added dimension to understanding a population’s health. The substantial literature that explores self-reported perception-based measures such as SRI and SRH with diagnosed clinical data highlights the need to better understand and interpret SRH and SRI [15–18]. This gap in our understanding of the nature of the relationship between SRI and SRH is particularly acute in conflict-affected settings.

In studies analysing both SRH and SRI, SRI is generally limited to being used as an indicator of the ‘robustness’ of SRH. When there is discordance between the two measures, this is frequently used to disregard the results of an analysis of SRH [19]. However, using SRI only as an indicator of the robustness of SRH means that analyses discount how SRI might be used to better understand how people view and understand their health. It is well established that two individuals reporting the same chronic illness (SRI) can rate their health (SRH) very differently [20]. It is therefore important to understand not only the relation between SRI and SRH, but also how, when and why they diverge across and within populations and contexts. Measures of health are political because they include or exclude specific information for use in management and policy; it is therefore essential that they are sensitive to the contexts in which they are applied [20]. The political nature of data on health is heightened in settings with political violence.

Whilst political and economic insecurity among Palestinians is positively associated with poorer objective and subjective health outcomes [21], studies have found that oPt women in general, and Gazan women in particular, are less likely to report ill health [22, 23].

Given the diverse socioeconomic and cultural conditions, disparate health systems in the oPt, it presents a unique opportunity to explore in greater depth aspects of the SRI-SRH relationship.

The aim of this study is to assess the socioeconomic determinants of three self-reported measures of health among women in the oPt—self-reported self-rated health (SRH) and two self-reported illness indicators (acute and chronic diseases) accounting for community and setting factors.

## Data and methods

We used the 2010 Palestinian Family Health Survey (PFHS), conducted by the Palestinian Central Bureau of Statistics (PCBS). The PFHS employed a multi-stage stratified sampling design to provide nationally representative demographic, health, and socioeconomic data for the Palestinian population living in the occupied Palestinian territory in 2010 (PCBS 2013). The survey is collected in collaboration with UNICEF and is based on the international standards of the Multiple Indicator Cluster Surveys (MICS) wave 4. Fieldwork was completed in August 2010 for the WB and October 2010 for the GS, with response rates of 90.5% and 94.8%, respectively. The sample is stratified by 16 governorates (5 in GS, 11 in WB) and 644 clusters (Primary Sampling Unit, PSU) (PCBS 2013). Within each of the clusters, 24 households were selected for the survey, yielding a sample of 15,355 households from which 19,509 women aged 15–54 were eligible for interview, of which 15,734 completed their interviews with a response rate of 74.2% (PCBS 2013).

For women aged 15–54 years, irrespective of marital status, data on SRI (chronic and acute) and SRH were collected [24]. Non-missing data on all covariates were included in the analyses, yielding a sample of n = 14,819 women aged 15–54 years with data on SRI (both chronic and acute) and SRH. Three binary dependent variables are analysed: self-rated health (SRH); self-reported acute illness (SRIa); and proxy self-reported chronic illness (pSRIc).

Self-Reported Health (SRH) is the most commonly used measure and it uses a 5 (or 6)-point Likert scale, with respondents rating their current health status from poor to excellent [25]. SRH is considered a subjective measure of health as it is based upon how a respondent ‘feels’ about their general health status at the time of interview (Johnson 2007). SRH is widely used in LMICs for four reasons: it is a single item measure; data are relatively easy to collect in a survey; SRH has been demonstrated to independently predict mortality and morbidity risks; and, it offers a measure in resource-poor settings where medical data are relatively difficult and expensive to obtain [18, 19, 26, 27].

In the 2010 PFHS, respondents were asked to evaluate their self-rated health on a 6-point scale (bad—acceptable—moderate—good—very good—excellent). In our analyses, women who rated their health as bad or acceptable or moderate were grouped as having a relatively poorer health status (20.8%; n = 3,449) and women who rated their health good or very good or excellent were grouped as having relatively good health (79.2%). Using such a grouping approach has been found to be effective in the analyses of comparative data because the results are less affected by context-specific perceptions of health [28]. Acute self-reported illness (SRIa) is a binary variable based on women’s self-reports of experience of a health problem in the 2 weeks preceding the interview (22.3% of the sample, n = 3,811). Chronic proxy/self-reported illness (pSRIc) data were collected differently and are based on reports by a member of the respondent’s household (who might not be the woman herself) of household members with a chronic disease that was diagnosed by a medical professional and for which regular treatment was being received at the time of interview. Chronic diseases reported include at least one of hypertension, diabetes, peptic ulcer, cardiac disease, cancer, renal disease, hepatic disease, arthritis (rheumatism), osteoporosis, thalassemia, epilepsy, asthma. back pain, endocrine levels. It is a limitation of the data that women’s own self-reports of chronic illness were not collected, and for our analyses we have to rely on these proxy report of chronic illness. pSRIc is a binary variable taking a value of 1 when the household respondent reports that a woman in the household, age 15–54, has a chronic disease. Exploratory analyses revealed no statistically significant differences in a male household roster respondent and a female household roster respondent reporting the incidence of a chronic illness among female household members age 15–54. Based on these proxy reports, 10.3% of sampled women are reported as having a chronic illness (n = 2,176). Previous research in LIMCs showed that proxy reports are as valid as self-reports although they are more robust if also physical reports are considered [29].

Determinants of these three binary dependent variables are analysed using logistic regression models. Prior to the estimation of the regression models, polychoric correlation coefficients, estimated using maximum likelihood estimates, are calculated to explore the bivariate nature between the three dependent variables which have shown to be predictive of each other in the literature.

Three separate logistic models for each health outcome have been estimated. These include demographic, socioeconomic and regional covariates alongside the remaining SRH/SRI variables to explore both the concordance between measures of SRI and SRH, controlling for the determinants of each, as well as the wider determinants of SRI and SRH the covariates describe.

Initially a single level logistic regression model is estimated, but given the multilevel nature of the determinants of health outcomes and the multi-stage sampling procedure employed in the PFHS two random intercepts are added sequentially to the model, an intercept for the sampling cluster and then an intercept for the governorate.

The sampling design of the PFHS is a multistage cluster sample, with households selected within geographic clusters. In order to maintain the independence of individual level observations (here, of the women) and prevent type I errors, a random intercept for Primary Sampling Units (PSU) was introduced in each model. For each model across the dependent variables (SRIa, pSRIc, SRH), a log-likelihood ratio test is conducted. A significant result of the log-likelihood ratio test shows that there is nesting if individual women within the intercept tested (here be it sampling cluster or governorate). Beyond the methodological need to introduce a random intercept for nested data to maintain the integrity of the assumptions of model, the intercept at the cluster level is a means to explore the importance of community effects on the three health measures, with each cluster identifying a neighbourhood in the oPt. Thus, in fitting the cluster random intercept we are able to not only control for nesting in the data, but also explore the amount of variation in each health outcome that is explained at the community level.

To further explore place effects in the data, an intercept for governorate is also introduced. Governorates are important as they reflect an administrative level at which health systems can differ within the oPt, both in policy and provision. Were the log-likelihood ratio test significant, an intercept for governorate will be included in the model. This intercept can maintain the assumptions of the model, accounting for the nesting of clusters within governorates but also enable the exploration of the amount of variation in the health measures explained at the community and governorate levels in the oPt. Data are weighted to control for the survey design and random intercept models run in Stata 13 [30].

In these models, covariates are introduced to analyse the determinants of SRH and SRI within the oPt, including demographic, socioeconomic and regional variables as well as data on pregnancy status, anemia and parity. These were included in addition to the socio-demographic variable as they could be factors of risk for specific health outcomes (e.g.: parity can have a positive effect on SRH). Demographic covariates included in the analysis are age and marital status. Age is included in the analysis as a categorical variable with 5 year age groups from 15–19 to 50–54. Marital status is introduced into the models as a categorical variable.

Socioeconomic status of women in the sample uses three variables: household wealth; a woman’s level of education; and, a woman’s employment status. Household wealth is taken as the status of the household in which the woman resides. The asset scores, based on Filmer and Pritchett (2001), are included in the model as a categorical variable of wealth quintiles determined from asset scores using Principle Component Analysis [31]. Education was used as a categorical variable reflecting the education system of the oPt (up to completed primary education, up to completed secondary education, some tertiary education). This decision was based on research from the oPt which shows that post-secondary education is important for health knowledge, and is an approach used by other authors [10].

Anaemia prevalence, pregnancy status and parity, self-reported by the respondents or proxies, were controlled for in the models, based on evidence showing that each independently increases the odds of reporting SRI and/or poor SRH [32]. A J-shaped relationship between parity and health (both physical and self-rated health), with nulliparous and highly parous women often having worse health outcomes, has been reported [33, 34]. A categorical variable for parity (nulliparous, parity of 1–3; 4–7 and highly parous (7+)) is introduced into the model to assess these relationships in the oPt with SRI and SRH. The SRH for pregnant women has been shown to vary particularly by obstetric problems during pregnancy [35]. In addition, maternal morbidity is also likely to impact SRI, as a covariate for pregnancy status is used in the models.

Region and locality variables were included in the models to examine the effect of place on the relationship between SRH and SRI. This variable is particularly important for a study of the oPt given the geographic variation in political violence and health systems.

For this analysis, four oPt administrative regions were defined: the GS, North WB, South WB and the Central WB (inclusive of the cities of Nablus and Bethlehem and the rural areas surrounding them). Within the oPt there are also three key types of locality—rural areas, urban areas and refugee camps. Both region and locality were included as fixed effects in the models. Routine checks for outliers, collinearity and leverage were performed.

## Results

### Sample description

The sample’s median age was 30 years. 63.1% of women in the sample are married, 34.6% are never-married, and 2.2% are divorced or widowed. As for place of residence 36.5% lives in GS, 17.6% in North WB, 14.8% in South WB and 31.1% in Centre WB. Intra-oPt differentials in socio-demographic and health measures are revealed. In general GS reports the largest percentage of poor households and the highest number of households with more than 8 children.

Moderate positive polychoric correlations (including chi squares) were found between reporting a chronic or reported health problem and a woman rating her health as ‘poor’ (Table 1). For women with a chronic illness, 57.9% rated their health as poor, whilst among women with no illness reported just 16.5% rated their health as poor (ρ = 0.563). For women who reported an acute health problem in the last 2 weeks, 43.8% rated their health as poor whilst of those not having reported health problems in the last two weeks only 14.3% rated their health as poor (ρ = 0.505). For women with acute health problems, nearly a quarter (23%) also have a chronic illness reported (ρ = 0.441). Thus bivariate relationships between SRI and SRH were as expected.

**Table 1:** Distribution of SRH, chronic and acute health problems oPt, PFHS 2010.

		Self-Rated Health (%)		*ρ*	Chronic Illness (%)		*ρ*	n
		Good	Poor		No	Yes		
Chronic Illness(%)	No	83.5	16.5	0.56				
	Yes	42.1	57.9					
Acute health problems (%)	No	85.8	14.2	0.50	93.5	6.5	0.4	11,223
	Yes	56.2	43.8		76.7	23.3		3,596
Total (%)		79.2	20.8	-	89.3	10.3	-	100
N		11,370	3,449		13,017	1,802		14,819

Reported anaemia is highest in the GS and Central WB (36.8% and 31.6%, respectively), compared to the North and South WB (17.7% and 13.8% respectively). The largest proportion of women who do not know their anaemia status is evident in the South WB (38.6% of women), and the reported levels of anaemia are likely and underestimate of true levels. Self-reported anaemia also varies by location and education in the oPt, higher levels of anaemia reporting are present among urban women and women with only primary level education.

## Regression results

The modelling strategy estimated a single level model, followed by a model with intercept for community (PSU) then a model for governorates. Log-likelihood Ratio tests results showed that both community and governorate intercepts significantly improved the model (Table 2). The final models presented are thus 3-level logistic regression models for the three separate dependent variables SRIa, pSRIc, SRH. Collinearity assessments showed no threat to model assumptions by including each respective health outcome in each model, nor indicated the need to remove any of the covariates.

**Table 2:** Log-likelihood ratio tests, multilevel models PFHS 2010.

Models Compared for Log-Likelihood Ratio Test	Acute	SRH	Chronic Illness
Community vs none	66.84***	95.68***	17.05***
Governorate vs none	139.1***	74.46***	9.89**
Community & Governorate vs none	166.4***	134.95***	22.66***

*** p<0.001

** 0.001<p<0.05

The multilevel logistic model regression results for each model underline the consistency between SRI and SRH in the oPt. Controlling for all other variables, a reported illness (chronic or acute) increases a woman’s odds of rating her health as poor (Table 3). A woman with SRIa is nearly 3 times more likely to rate her health as poor than a woman who has not reported acute health problems (OR 2.93, p<0.001). This concordance is also present when looking at pSRIc; a woman with a proxy report of chronic illness is over twice as likely to rate her health as poor compared to women who have not been reported as having a chronic illness (OR 2.83, p<0.001), controlling for all other variables.

**Table 3:** Three-level logit models regression results for chronic, reported illness and SRH among women age 15–54 in the occupied Palestinian territories, 2010.

		Model 1	Model 2	Model 3	% of sample (weighted)
FIXED		Acute	SRH	Chronic	
Education	Up to completed primary	1.09	1.65***	1.62***	60.92
	Secondary	1.08	1.33***	1.23	16.26
	Some tertiary	1	1	1	22.82
Region	Gaza Strip	1	1	1	36.46
	North West Bank	2.16***	1.70***	1.17	17.65
	South West Bank	1.14	1.56	1.03	14.80
	Centre West Bank	1.66***	1.60**	1.18	31.09
Locality	Camp	1	1	1	10.13
	Rural	0.84	0.79*	0.62***	17.05
	Urban	0.84	0.91	0.74**	72.81
Household Wealth Quintile	Poorest	1.32***	1.86***	1.31*	18.10
	Poorer	1.16*	1.73***	1.18	19.55
	Middle	1.11	1.44***	1.21	20.99
	Richer	1.03	1.24**	1.11	20.64
	Richest	1	1	1	20.73
Parity	0	1	1	1	36.21
	1 to 3	0.99	1.63*	1.82	24.66
	4 to 7	1.12	1.77	2.02	30.24
	More than 7	1.21	1.87*	1.73	8.89
Age	15–19	1	1	1	23.36
	20–24	1.24	1.46**	1.14	18.64
	25–29	1.40**	1.95***	2.39**	14.70
	30–34	1.41**	2.37***	3.97***	12.57
	35–39	1.44**	2.83***	7.042***	10.54
	40–44	1.43**	3.749***	13.845***	08.62
	45–49	1.39*	4.05***	22.2.438***	06.77
	50–54	1.18	5.19***	41.251***	04.80
Employment Status	Unemployed	1	1	1	65.60
	Employed	1.07	0.75***	0.942	09.42
	Student	1.05	0.94	0.810	24.98
Marital Status	Never Married	0.60*	1.14	1.682	34.66
	Divorced/Widowed	1.804	3.258**	1.78	02.20
	Married	1	1	1	63.15
Pregnancy status	Not	1	1	1	88.79
	Yes	0.847*	1.273**	0.646**	08.74 02.47
	Do not know	0.616	0.396*	0.711	
Anaemia	No	1	1	1	89.81
	Yes	2.159***	2.005***	1.683***	06.86
	Don’t Know	1.168	1.809***	0.870	03.33
Chronic Illness	No Yes	1 2.239***	1 2.833***		89.71 10.29
Reported health problems	No Yes		1 2.930***	1 2.224***	77.70 22.30
SRH	Average to Good Moderate to Poor	1 2.942***		1 2.767***	79.21 20.79
Constant		0.098***	0.017**	0.004***	-
RANDOM:					
Cluster-level		0.449***	0.403***	0.349***	-
Governorate		0.201***	0.252***	0.157***	-
N		14819	14819	14819	100.00

***p<0.001

**p<0.01

*p<0.05

Looking at the multilevel structure, whilst there is a concordance between both chronic and acute SRI measures with SRH, there is divergence in the explanatory patterns for reporting illness or poor SRH among women aged 15–54 in the oPt. Self-reports of anaemia are significantly associated with increased odds of both chronic and acute SRI, as well as with poor SRH. The intraclass correlation coefficients in Table 3 are used to show the correlation within the levels specified in the multilevel models. Although marginal (Table 4), governorates significantly explain a proportion of the variance in all three health outcomes (Table 3). For SRIa 1.1% of the variance is explained at the governorate level, compared to 0.7% for chronic illness. For SRH, 1.8% of the variance in reporting poor health or otherwise among women in the oPt is explained at the governorate level. At the community level (PSU/Cluster), within governorates, a greater proportion of the variance in health outcomes is explained– 6.8%, 6.4% and 4.3% for reported health problems, SRH and chronic illness, respectively. This variance could be explained by a lower effect of the health systems but more significant of the community level services being affected often by blockades and embargos.

**Table 4:** Intraclass correlation coefficients for 3-level model (women within communities within governorates) from multilevel models oPt 2010.

Dependent Variable	Acute *(95% Confidence IntervaI)*	SRH *(95% Confidence IntervaI)*	Chronic Illness *(95% Confidence IntervaI)*
Governorate	0.011 *(0*.*005–0*.*028)*	0.018 *(0*.*007–0*.*044)*	0.007 *(0*.*002–0*.*029)*
Community within Governorate	0.068 *(0*.*052–0*.*089)*	0.064 *(0*.*046–0*.*089)*	0.043 *(0*.*025–0*.*073)*

Regionally, there are differences in the explanatory patterns for the different reported health measures. However, across all four regions, no difference is found in the odds of a woman reported as having a chronic illness, holding all other variables constant (Table 3).

The community level variable has no significant effect on the odds of reporting illness in the last 2 weeks. However, locality (e.g.: camp/rural/urban) is significantly associated with both reporting a chronic illness and poor SRH. Women living in camps are more likely to report poor SRH and chronic illness compared to women in rural areas (OR 0.620, p<0.001 for rural women), and are also more likely to report a chronic illness compared to women living in urban areas (OR 0.745, p<0.01 for urban women). The results show pervasive regional differences in SRI and SRH in the oPt; some variation in health measures are explained at the governorate and community levels and there are clear regional and locality differences in SRI and SRH (Table 3).

Household wealth has the most consistent effect across all three health measures; with women in the poor and poorest wealth quintiles being more likely to report illness or poor health than wealthier groups. For SRH, there is a consistent wealth gradient, with the poorest women 1.9 times more likely to rate their health as poor compared to women in the richest quintile. For chronic and reported acute health problems, women are 1.3 times more likely to self-report incidence of either illness in the poorest quintile compared to the richest quintile. However, for pSRI, being in the richest wealth quintile is only comparatively protective against illness compared to women from the poorest households. Women in the richest wealth quintile are no more likely to report not having an acute or chronic illness compared to all other women, suggesting an attenuated effect of wealth on women’s health in the oPt in all but the poorest households (Table 3).

Employment status only has a significant effect for SRH, with employed women less likely to rate their health as poor compared to unemployed women (OR 0.752, p<0.001). For SRI, both acute and chronic, there is no difference between employment categories (Table 3).

Education level has no effect on the odds of SRIa, controlling for all other variables. However, women who have complete primary education are more likely to report both poor SRH and pSRIc compared to those with some tertiary education. Women with some tertiary education are also less likely to report a chronic illness than women who have secondary education. In general, the results are consistent with higher socioeconomic status being protective against poor health measures, but this varies between SRI and SRH measures.

The results show an expected age gradient in SRI and SRH, with older women having higher odds of reporting poor health compared to young women aged 15–19. Never married women, are 40% less likely to have a SRIa than married women, although never married women reported no significant differences in chronic illnesses and SRH compared to married women. Women who are divorced are 3.2 times more likely to rate their health as poor compared to married women, holding all other variables constant. Marital status, for women, has no effect on the likelihood of a chronic illness being reported (Table 3).

Controlling for all other variables, parity is only a statistically significant predictor of SRH. Women with either 1–3 children or more than 7 children are more likely to report poor SRH compared to their nulliparous peers.

Being pregnant increases the odds of a woman rating her health as poor, compared to those who are not pregnant (OR 1.273, p<0.01). However, being pregnant reduces the odds of reporting either SRIa or chronic illness (OR 0.847, p<0.05 and OR 0.646, p<0.01 respectively). Not knowing current pregnancy status reduces the odds of reporting SRH (OR 0.396, p<0.05). Whilst there are regional and socioeconomic differentials in the odds of reporting poor health measures, the explanatory patterns vary by health measure (Table 3).

## Discussion

This study assessed the socioeconomic determinants of three self-reported measures of health among women in the oPt: self-rated health; self-reported illness for acute illness; and, self-reported illness for any chronic disease. Analyses of the socioeconomic determinants of these measures in one context contribute to an understanding of the relation between SRI and SRH, whilst accounting for context-specific factors.

Our analyses showed a concordance between SRI and SRH health measures with significant differences in explanatory patterns across the oPt by region and socioeconomic status. Not only were there regional differences in health in general across the oPt, but there were also regional differences in measures of subjective and objective health. Women from the GS reported lower levels of self-reported and self-rated poor health outcomes. Gazan women’s reports of better health (both objective and subjective) were at odds with their relatively poorer health infrastructure, living conditions, nutrition and socioeconomic status due to the severity of occupation violence in Gaza. This finding highlights the importance of understanding context for both objective and subjective measures of health. Relative to other regions, the Gaza Strip experienced the most extreme consequences of the ongoing conflict in the oPt. Severe restrictions of the movement of people and goods, the economic and physical ramifications of the conflict has left the region with the poorest health system infrastructure in the oPt [13]. Cultural, structural and psychosocial resilience have been reported in contexts of prolonged conflict [36]. Qualitative studies from the Gaza Strip point to Gazan women focusing on the wider concerns of their family above and beyond the ongoing conflict and their own health [37]. For women in the Gaza Strip, even if their health and living conditions are suboptimal, they were less likely to rate their health as poorer than Palestinian women from the West Bank, because they consider their health as relatively less significant compared to that of their family and their wider community enduring dire conditions. That is, their rating is likely relative to the suffering they experience around them, and to other factors inhibiting human functioning above and beyond health. Lower living standards, poorer nutrition and more conflict-related injuries in addition to an overburdened health system all contribute to the concentration of reported health problems, chronic disease and an associated evaluation of individual health as poor among the Gaza Strip population.

Employed women, relative to unemployed women were less likely to report poor SRH. Women in employment tended to have better health compared to those not in the labour force, explained as a combination of a selection effect and higher levels of life satisfaction [38]. Being never married was associated with a lower likelihood to report SRIa and poor SRH. This could be attributable to lower levels of never-married women’s engagement with the health system in general because of the heavy emphasis on maternity-related care services for women’s health in oPt. Never-married women who are not mothers were much less likely to interact with health services in general, not least because of health professionals’ assumptions about health-related behaviours of never-married women. Never-married Palestinian women face social status disadvantage and barriers to health care. In a study of the causes of death of all women in 2000–2001 in oPt, the authors found that barriers to accessing health care by never-married women may be related to increased mortality [39]. Divorced and widowed women were more likely to report poor SRH; this could be explained by relative levels of poverty among this sub-group in general [40]. Widowed or divorced women who do not possess their own money or lack control over family resources become highly dependent on their families for support; being divorced is highly stigmatized in the oPt context [41]. In analyzing the regional effects on health in the oPt, the role of socioeconomic conditions have been found to be important. Wealth was protective for both the two SRI measures and poor SRH, although education and employment status are not uniformly significant across the four regions of the oPt.

The results for SRI also show that access to improved health infrastructure remains important for women’s health in the oPt. In camps, women were more likely to report a chronic illness, compared to women from either urban or rural areas. There were two possible, non-exclusive, explanations for this finding. Firstly, it is possible that there is a higher prevalence of chronic illness in refugee camps compared to urban and rural areas. Living conditions in camps, particularly those with large population densities in areas of chronic exposure to political violence, can be conducive to increased levels of stress, poverty and poor nutrition that have been shown to increase the likelihood of developing a chronic disease [42]. Research on diabetic patients from GS refugee camps showed that the impact of diabetes on health-related quality of life is worse than diabetic patients living elsewhere, attributed to the living conditions and increased stress of life in the camps [43]. It is also possible there are greater odds of women in refugee camps reporting chronic illness due to greater access to health care provided by the United Nations Relief and Works Agency for Palestine Refugees (UNRWA), and thus actual diagnoses. Better access might also mean that women in camps have more health literacy and are more likely to seek healthcare. Although there is no information directly related to chronic illness and health care access in the PFHS, among women with SRIa in the last two weeks, women in camps are significantly more likely to access health care (F 3.18; p<0.05).

The finding that women in camps reporting better health than elsewhere supports the argument that, with chronic illness reporting, we are likely seeing higher rates due to greater access to health care, better health knowledge and also a higher rate of diagnosis of chronic illness and not necessarily just a greater incidence in refugee camps. The importance of health infrastructure and health outcomes in the oPt is also supported by the significance of the random intercept at the governorate level. In particular, with respect to government-run health services, health system planning and management is mainly centralized by the Ministry of Health in the oPt, but there is some variability to local directorate control and management at the governorate level, giving rise to further variations in health provision and access at the governorate level (WHO 2006). More specifically the Palestinian Authority’s distribution of health services and personnel is unevenly distributed by governorate in relation to population and also skewed towards hospital care, which accounts for the bulk of the national health budget [44].

Women in camps were also more likely to rate their health as poor compared to rural women, which is at odds with relatively better health care access but might be explained by better lifestyle and nutrition in rural areas, with lower levels of psychosocial stress from living in areas with high population density, in addition to perhaps better health knowledge given better access to health care services in the camps. Again, this result highlights the concordance between SRIc and SRH. Across areas in the oPt, there is a diverse tapestry of factors that affect women’s health, and their importance varies across regions and localities.

When we considered both socio-cultural aspects and access to health care, the fact that Gazan women had lower odds of reporting health problems, contrary to what would be expected given the living conditions, poverty and population density in the Gaza Strip, seems understandable. In addition to perhaps Gaza’s women rating their health in relation to others in their community and the dire context in which everyone lives making health complaints perceived as insignificant, increased inequality has been found to be associated with higher rates of poor SRH, and Gaza has the lowest level of inequality in the oPt (PCBS 2015). Thus despite higher levels of psychosocial stressors, poor living conditions, risk of injury and disease in GS, lower levels of inequality may well lead to lower levels of SRI and poor SRH.

## Conclusions

Our analyses show that in the oPt women’s health is determined by a diverse range of factors, including regional, socioeconomic, demographic and cultural factors. There is a concordance between SRI and SRH measures in the oPt, suggesting that both measures are capturing elements of an underlying concept of health. However, the differences between SRI and SRH highlight the importance of elucidating and understanding more subjective and objective measures of health.

Both subjective and objective health measures should continue to be included in future health surveys in order to better understand how local populations perceive and feel about their wellbeing. There is a need for more detailed data on reported health problems, with differentiation in terms of severity and whether acute or chronic. Current, routine data collection does not permit disaggregated analyses by severity of reported health problems. Finally, there is a need for more qualitative research to better understand how health is understood in diverse contexts.

Moving forward policies at national and international level in this area will need to focus more on the needs of women in a more holistic approach which includes pre and post reproductive life. This will need to include the mental and physical wellbeing in the aftermath of post-childbearing, including peri-menopausal healthcare. Mental health as the SRH results show, is key in understanding the stigma and feeling of worthiness that follows the move of the centre of attention from being the child bearer to simply a wife in the household once the children have left. Access to diagnostics as well as to overall health care needs to be approached in a lifecourse manner in particular in conflict settings where barriers to access are further heightened. Community health workers for example could be directed in this respect would be of great use both in terms of outreach and in terms of local understandings of current needs.

This study highlights the need for a greater understanding of context when collecting health-related data in settings such the oPt affected by political violence. It also supports the understanding that, among Palestinian women at least, it is both place and subjective perspective that are important for not only the conceptualization of health but also its reporting. Finally there is a need for a greater understanding of women’s health needs over the lifecourse moving beyond narrow foci on ‘reproductive health’ and ‘health of mothers’. Without a deeper understanding of this we are not able to move forward in trying to meet peoples’ health needs.
