# Correction: Women's health in the occupied Palestinian territories: Contextual influences on subjective and objective health measures

**DOI:** 10.1371/journal.pone.0191540

**Published:** 2018-01-16

**Authors:** The PLoS ONE Staff

The affiliation for the second author is incorrect. Tiziana Leone is not affiliated with #1 but with #2 Department of International Development, LSE, London, United Kingdom. The publisher apologizes for this error.

## References

[1] Bates K, Leone T, Ghandour R, Mitwalli S, Nasr S, Coast E, et al (2017) Women’s health in the occupied Palestinian territories: Contextual influences on subjective and objective health measures. PLoS ONE 12(10): e0186610 https://doi.org/10.1371/journal.pone.0186610 29077709 10.1371/journal.pone.0186610 PMC5659643

